# Whole-genome sequencing of *Fusarium oxysporum* K326-S isolated from tobacco

**DOI:** 10.1128/mra.00319-25

**Published:** 2025-09-10

**Authors:** Yahui Yang, Yingjie Liu, Yong Peng, Jian Chen, Pengjun Xu, Yonghui Zhang

**Affiliations:** 1 Key Laboratory of Tobacco Pest Monitoring Controlling & Integrated Management, Tobacco Research Institute of Chinese Academy of Agricultural Sciences, Qingdao, China; 2 Staff Development Institute of China National Tobacco Corporation, Zhengzhou, China; 3 Luzhou City Company of Sichuan Province Tobacco Company, Luzhou, China; University of California Riverside, Riverside, California, USA

**Keywords:** *Fusarium oxysporum*, genome

## Abstract

This report presents the whole-genome sequence of *Fusarium oxysporum* K326-S (*F. oxysporum* K326-S), a soil-borne pathogenic fungus. Its genome is 51,143,096 bp with 1,153 scaffolds and 17,272 coding genes, providing valuable insights for future disease prevention and control research.

## ANNOUNCEMENT

Root diseases increasingly threaten plant health, highlighting the importance of managing soil-borne pathogens (1, 2). Whole-genome sequencing of root-associated fungi provides critical insights for disease control (3). Here, *Fusarium oxysporum* K326-S was isolated from *Nicotiana tabacum* L. (K326) roots showing browning and wilting symptoms, collected at the Southwest Experimental Base of the Chinese Academy of Agricultural Sciences (27°49′48″N, 102°22′12″E). Root segments (~1 cm) were washed, surface-sterilized (75% ethanol, 30 s; 1% sodium hypochlorite, 1 min), rinsed in sterile water, and cultured on PDA at 28°C for 5 days. The isolate was identified based on morphological features and ITS sequencing (ITS1/ITS4), showing >99% identity to *F. oxysporum* K326-S reference sequences in NCBI. BLAST analysis against the FusariumID database also showed 99% identity to *F. oxysporum* isolate LuC1-1-48 (GenBank: PP864100.1), though forma specialis could not be determined. The isolate, designated *F. oxysporum* K326-S, is maintained in the culture collection of the Chinese Academy of Agricultural Sciences.

*F. oxysporum* K326-S was cultured in potato liquid medium at 28°C with shaking (200 rpm) for 3–5 days, reaching the mycelial stage. Genomic DNA was extracted using a fungal genomic DNA extraction kit (T07-100, TSINGKE Biotech, China), following the manufacturer’s magnetic bead-based protocol. DNA was sheared to ~15 kb using a g-TUBE, and SMRTbell libraries were prepared according to PacBio protocols (4). Sequencing was performed on a PacBio Sequel II platform using HiFi reads (5). A total of 2,990,184,144 bases were generated. After quality control using fastp v0.20.0, 2,801,252,197 clean bases remained, with Q20 and Q30 values of 99.18% and 97.16%, respectively (6). Clean reads were assembled using HiFiAsm v0.19.8-r603 (7), producing 1,153 scaffolds with a total length of 51,143,096 bp, and a maximum scaffold length of 1,078,849 bp and N90 of 20,426 bp. The estimated sequencing depth was approximately 84×, calculated as the ratio of total clean bases to assembled genome size. Repetitive sequences were masked using RepeatMasker v4.1.4 (8) with the default RepBase library and the species parameter set to “fungi.” Genome completeness was evaluated using BUSCO v5.4.4 (9) with the ascomycota_odb10 lineage data set. The assembly showed a high completeness of 98.9%, with only 0.3% fragmented and 0.8% missing BUSCOs, indicating a highly complete fungal genome. Gene prediction was performed using MAKER2 v2.32 (10) (with Augustus v3.5.0 (11), GeneMark-ES v4.71 (12), and SNAP (13). tRNAs and rRNAs were identified using tRNAscan-SE v2.0.12 (14) and barrnap v0.9 (15). Functional annotation was done via BLAST+ v2.3.0 (16) and DIAMOND v0.8.35 (17) against NR (18), Swiss-Prot (19), Pfam (20), eggNOG (21), GO (22), and KEGG (23). Default parameters were used for all software unless otherwise specified. A total of 17,272 coding genes were predicted, along with 310 tRNAs and 63 rRNAs. The assembled genome of *F. oxysporum* K326-S expands the available genomic resources for the *F. oxysporum* species complex and provides a foundation for future research.

## Data Availability

Accession numbers are as follows: for genome assembly, GenBank JBPBVU000000000; for raw reads, SRA SRR32787983; BioProject, PRJNA1238792; and BioSample, SAMN47479106. The genome annotation file is hosted on Figshare: doi.org/10.6084/m9.figshare.29447774.
